# Sleep Treatment Education Program for Cancer Survivors: Protocol for an Efficacy Trial

**DOI:** 10.2196/60762

**Published:** 2024-11-28

**Authors:** Briana L Bice, Alexis L Michaud, Katherine G McCormick, Eva M Miklos, Indiana D Descombes, Cheryl Medeiros-Nancarrow, Eric S Zhou, Christopher J Recklitis

**Affiliations:** 1 Perini Family Survivors' Center Dana-Farber Cancer Institute Boston, MA United States; 2 Department of Psychology Suffolk University Boston, MA United States; 3 Harvard Medical School Boston, MA United States

**Keywords:** insomnia, mood, cancer survivors, online interventions, protocol, cognitive behavioral therapy, CBT, cognitive behavioral therapy for insomnia, CBTI, digital health, sleep disorders, sleep treatment education program, STEP-1

## Abstract

**Background:**

Cancer survivors are at increased risk for chronic insomnia, even years after treatment completion. As insomnia is associated with a variety of long-term health consequences, access to insomnia treatment is critically important for the survivor population. Cognitive behavioral therapy for insomnia (CBTI) is the recommended first-line treatment for insomnia but remains largely unavailable to survivors. Treatment barriers include geographic limitations, a shortage of trained providers, and demanding treatment regimens. Designed with these limitations in mind, the Sleep Treatment Education Program (STEP-1) delivers components of CBTI in a low-intensity educational intervention delivered online.

**Objective:**

This is a phase II pilot randomized controlled trial. The primary aims are to test the efficacy of STEP-1 to improve (1) insomnia symptoms and (2) mood in cancer survivors compared to a control condition. The secondary aims will (1) explore participant factors associated with clinically significant response, (2) evaluate acceptability of the control intervention, (3) explore feasibility of delivering individualized coaching sessions for participants who do not have a significant response to STEP-1, and (4) describe participants’ satisfaction with STEP-1 and suggestions for improvement.

**Methods:**

This 2-arm randomized controlled trial enrolled 70 off-treatment cancer survivors aged 40-89 years with clinically significant insomnia. Participants are randomized to receive either the STEP-1 intervention or control condition (relaxation education); interventions are delivered in one-on-one, synchronous, virtual videoconference sessions by trained interventionists. The STEP-1 intervention presents educational information on the development of insomnia after cancer and offers suggestions for improving insomnia symptoms based on the CBTI elements of sleep hygiene, stimulus control, and cognitive restructuring. With the interventionist, participants review the suggestions and develop a personalized sleep action plan for implementation. The relaxation education session provides information on the potential benefits of relaxation and how to independently access online relaxation exercises. The Insomnia Severity Index is used to measure insomnia symptoms, and the Profile of Mood States Short Form is used to measure mood at baseline and 4 and 8 weeks after intervention. The primary end point is change in the Insomnia Severity Index score at 8 weeks, and the secondary end point is change in mood symptoms (Profile of Mood States Short Form) at 8 weeks.

**Results:**

This trial was funded in July 2022. Enrollment and data collection began in February 2023 and concluded in October 2024, with 70 participants enrolled. The analysis will begin in fall 2024, and the results are expected in winter 2025.

**Conclusions:**

Trial results will determine if STEP-1 effects go beyond those that could be attributed to placebo and other nonspecific treatment factors. Should results support the efficacy of STEP-1 to improve mood and insomnia symptoms, we anticipate developing efficacy and implementation trials of STEP-1 in larger and more diverse samples.

**Trial Registration:**

ClinicalTrials.gov NCT05519982; https://clinicaltrials.gov/study/NCT05519982

**International Registered Report Identifier (IRRID):**

DERR1-10.2196/60762

## Introduction

Intensive treatments that improve cancer outcomes place survivors at high risk for developing medical and psychosocial late effects [1-5]. While insomnia may typically be considered a minor symptom in the larger cancer context, it frequently develops into a debilitating chronic condition related to significant health problems. In the general population, indirect economic costs of untreated insomnia exceed US $100 billion in the United States per year, primarily from diminished workplace performance, increased use of health care, and increased accident risk [6,7]. As many as 1 in 4 cancer survivors experience clinically significant insomnia even 10 years after cancer treatment has ended [8,9]. If untreated, insomnia is associated with heart disease, obesity, hypertension, diabetes, depression, and anxiety [4,10-18]. Survivors are already vulnerable to many of these same health conditions due to their cancer treatment history; therefore, in order to maintain their overall health, access to effective insomnia treatment is critically important.

Cognitive behavioral therapy for insomnia (CBTI) is a well-established and empirically supported treatment. A systematic review found that CBTI given to cancer survivors “provides significant, lasting improvement” [19]. Even compared to pharmacotherapy, multiple randomized trials have demonstrated that it is the most effective long-term insomnia treatment [20-22].

Despite compelling evidence, growing populations of cancer survivors who need it find this treatment largely unavailable [23]. Fewer than one-third of the most well-resourced cancer centers in the United States provide CBTI for patients or survivors [24]. To make effective insomnia treatment available to survivors, existing barriers must be resolved, including the shortage of trained CBTI providers and the need for more efficient and less burdensome treatments than standard CBTI, which have limited uptake and dropout rates of 20% to 40% [25-28].

To address these challenges and deliver effective insomnia treatment to cancer survivors, we have developed the Sleep Treatment Education Program (STEP-1). The STEP-1 intervention includes (1) core CBTI components, (2) answers to survivors’ frequently asked questions about sleep and CBTI, and (3) a written sleep action plan to promote adherence.

The STEP-1 intervention is brief, low cost, and low intensity and targets survivors’ specific needs and experiences. Delivered in a single virtual videoconference session, STEP-1 does not require travel to a specialized site for insomnia treatment and is available for survivors to access anywhere. STEP-1 provides guided behavioral planning to help survivors make informed choices to manage their insomnia. This self-management approach aims to support survivors’ autonomy and avoid a prescriptive stance [29]. In a single-arm pilot study, STEP-1 was found to significantly reduce insomnia symptoms in cancer survivors [30]. As the next step in this line of work, the efficacy of the STEP-1 intervention will be evaluated in a larger sample and compared to a control intervention in the randomized controlled trial described here.

The primary aims of the study are (1) to determine the efficacy of STEP-1 to reduce insomnia and (2) to improve mood symptoms in survivors compared to a control condition. Specifically, we hypothesize that insomnia symptoms (primary outcome) and mood (secondary outcome) will improve significantly in STEP-1 participants relative to control condition participants (primary aim). Secondary aims are (1) to explore participant and program adherence factors associated with clinically significant response to STEP-1, (2) to evaluate the acceptability of the control intervention, (3) to explore the feasibility of delivering individualized coaching sessions for participants who do not have a significant response to the STEP-1 intervention, and (4) to describe participants’ satisfaction with STEP-1 and their suggestions for improving the intervention.

## Methods

### Study Design

The STEP-1 intervention is being evaluated in a pilot randomized clinical trial, guided by the Obesity-Related Behavioral Intervention Trials (ORBIT) model [31]. Although originally developed by obesity researchers, the ORBIT model has been adapted for the development and early testing of behavioral interventions of all kinds [31-33]. Participants are randomized (1:1) to receive either (1) the STEP-1 intervention or (2) relaxation education (enhanced usual care control condition). STEP-1 and relaxation education are each delivered in a single online session via synchronous videoconference. Outcomes are assessed at baseline and again 4 and 8 weeks postbaseline. Participants will be recruited from treatment centers, advocacy groups, and social media and will complete all study activities remotely online. Of note, a modified version of STEP-1 adapted to address the needs of young adult survivors is currently being evaluated in a separate trial using similar methods [34].

### Participants

Participants will be 70 off-treatment survivors aged 40-89 years. To be eligible for enrollment, participants were diagnosed with cancer (excluding nonmelanoma skin cancer) at least 1 year prior, have received no cancer therapy (excluding chemoprevention) in the past 4 months, and have no additional cancer therapy planned. Participants must be able to read and write in English, have access to the internet on a daily basis, and have significant insomnia as evidenced by an Insomnia Severity Index (ISI) score greater than or equal to 12 [35]. Survivors who have specific health conditions, medications, or past insomnia treatments that may affect their sleep or response to STEP-1 are excluded; for participant inclusion and exclusion criteria, see Table 1.

**Table 1:** Study inclusion and exclusion criteria.

Category and criteria			Rationale
**Inclusion criteria**			
	**General**		
		Age 40-89 years Able to read and write in English	Enroll off-treatment survivors able to participate in the intervention currently available only in English.
	**Health issues**		
		History of a cancer diagnosis (except nonmelanoma skin cancer) ≥1 year prior No active cancer therapy (excluding chemoprevention) in the past 4 months, and no further therapy planned	Enroll off-treatment survivors able to participate in the intervention currently available only in English.
	**Internet access**		
		Regular access to the internet on a daily basis	To ensure participants have adequate ability to complete online study.
	**Sleep problems**		
		Significant insomnia, as evidenced by an Insomnia Severity Index score ≥12	To test this intervention in individuals with clinically significant insomnia.
**Exclusion criteria**			
	**General**		
		Any impairment (eg, sensory and cognitive) that interferes with the ability to complete all study measures independently	Exclude individuals not able to independently complete the study procedures.
	**Health issues**		
		Report ever being diagnosed with bipolar disorder Report ever being diagnosed with a seizure disorder or have experienced a seizure in the past 12 months	Exclude individuals for whom behavioral treatment for insomnia may require modification due to medical conditions [36,37].
	**Sleep problems**		
		Intention to adjust (decrease or increase) the use of any prescribed or over-the-counter medications taken to decrease insomnia during the study period Survivors who report being diagnosed with sleep apnea who are not receiving recommended medical treatment Survivors who report suspected sleep apnea who have not completed an evaluation by a sleep specialist Survivors who report their usual bedtime is not between 5 PM and 5 AM Employment that involves irregular sleep patterns, such as shift work or frequent long-distance travel across time zones, or employment in a position that could impact public safety	Exclude individuals whose sleep symptoms may require significant modifications in behavioral treatment for their insomnia [38,39].
	**Prior insomnia treatment**		
		Prior participation in a research study, which provided an educational or behavioral intervention for insomnia. Prior participation in a behavioral treatment or patient education program for insomnia delivered at the study site. Participation in behavioral or educational interventions for insomnia in the 2 years prior to enrollment (including synchronous and asynchronous online insomnia programs).	Enroll only participants naïve to the insomnia educational programs and behavioral treatments.

### Recruitment

All procedures and materials for recruitment are designed to allow survivors to self-refer to the study. These materials include a study information sheet (Multimedia Appendix 1), outreach letters and emails, informational postcards, and brief study descriptions suitable for patient-facing materials distributed by patient support and advocacy groups. For survivors interested in learning more about the study, all materials include phone, email, and web contact information. Examples, including a brief informational video, can be seen on the study website: StepForSleep.com.

Several methods are used for distribution of these materials to potentially eligible survivors. At our cancer center, study information postcards have been placed in waiting areas. Around the hospital, study information appears on patient-facing screens (eg, in the cafeteria and waiting areas). The study team meets with clinical providers who are informed of the study and given recruitment information and materials to share with patients. Several clinical teams have collaborated with the study team to send recruitment information directly to their potentially eligible patients. Additionally, center programs that serve supportive care needs are also asked to include study information in their newsletters, emails, or social media posts. Following similar procedures, study information and materials have been shared with providers at several other cancer centers and with patient support and advocacy groups (regionally and nationally) for distribution to their patients or members. These informational materials are also posted on research portals and selected social media sites (eg, Facebook; Instagram; Reddit; and X, formerly known as Twitter).

### Procedures

#### Screening and Scheduling

Survivors interested in STEP-1 first contact the study team via email or phone call. Any preliminary questions about the study are answered, and survivors are provided with the study information sheet (Multimedia Appendix 1), which includes details on the study procedures and aims. Survivors who are interested in participating are scheduled for screening via videoconference. The screening meeting is conducted by a research assistant (BLB, KGM, EMM, and IDD) who answers any additional questions survivors may ask about the study and administers the eligibility screening. Eligible survivors are invited to schedule a STEP-1 intervention session, and ineligible survivors are provided with a resource guide [40] for finding help with insomnia or other sleep issues. Any questions or concerns in this process are referred to the study’s principal investigator (CJR) for clarification and final determination of eligibility.

#### Intervention Session

**Figure 1:**
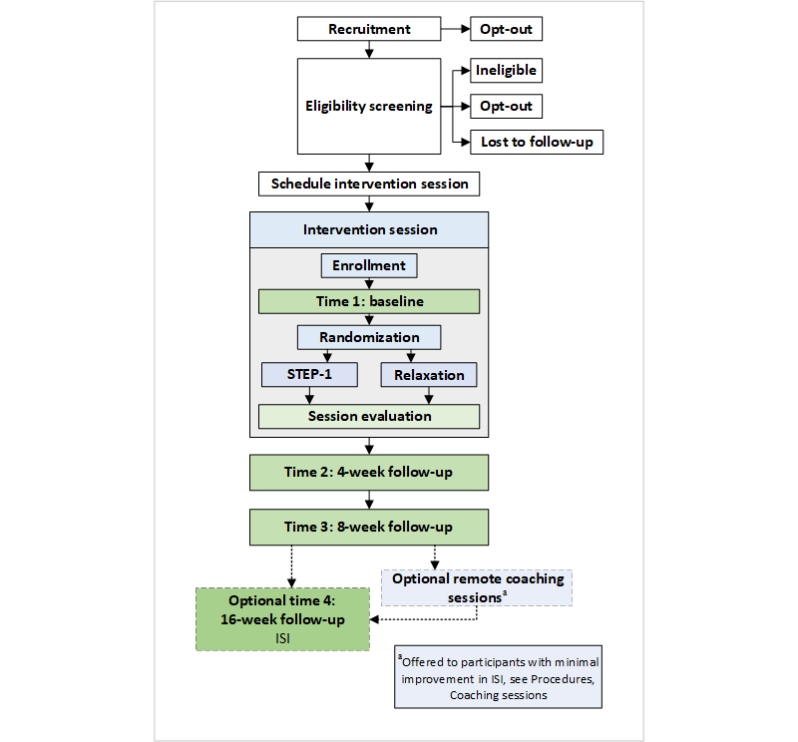
Participant flow diagram. ISI: Insomnia Severity Index; STEP-1: Sleep Treatment Education Program.

The intervention session is delivered in a single, synchronous, one-on-one videoconference session. Eligible participants complete the institutional review board (IRB) approved online consent procedure and a baseline survey before being randomized to receive the control condition or STEP-1 intervention (Figure 1). Following the intervention, participants are scheduled for follow-up contact.

#### Baseline Survey

Consented participants complete a baseline survey directly in the web-based Qualtrics survey platform [41]. Participants enter data directly into Qualtrics with study staff offering technical support as needed but not collecting data directly from participants.

#### Randomization

After the baseline survey is complete, participants are randomized using Sealed Envelope [42], a commercially available digital application for randomizing patients in clinical trials. Participants are randomized (1:1) to either (1) STEP-1 or (2) an enhanced usual care control condition (relaxation education). The study arms are balanced by age group; randomization, with a block size of 4, is stratified by those between 40 and 64 years versus 65 and 89 years. Once randomized, participants are informed and immediately receive their assigned intervention session via videoconference.

#### STEP-1 Intervention Delivery

STEP-1 is delivered in individual sessions by trained interventionists during a single 75-minute synchronous videoconference session using HIPAA (Health Insurance Portability and Accountability Act) compliant technology [43-45]. Interventionists deliver STEP-1 using a slide deck of 42 slides and following a structured outline, with copies of session materials (eg, presentation slides and sleep action plan template) provided to participants by email. During the session, the participant can see and hear the presenter, view the presentation slides, and ask questions in real time. After introducing educational information based on Spielman’s model of insomnia [46] and the association of insomnia with cancer, the interventionist presents suggestions for improving survivors’ sleep based on CBTI components of sleep hygiene, stimulus control, and cognitive restructuring [19,27,47-49]. For ease of comprehension, this material is presented in 4 sections: addressing lifestyle issues (eg, limiting alcohol and stimulants), sleep environment (eg, avoiding bed for nonsleep activities), sleep timing (eg, setting a regular wakeup time), and managing expectations and challenges (eg, sleep worry and physical symptoms), with the underlying rationale and potential benefit for each suggestion provided. After each of the 4 sections, the participant is asked to review the suggestions presented and complete their sleep action plan template, with the interventionist supporting them to record their current sleep practices and behavioral changes they intend to make. Action plans of this kind are commonly used in behavioral self-management interventions [29] and have been shown valuable in promoting successful behavior change [50,51]. Information about how the COVID-19 pandemic may exacerbate sleep problems and what aspects of STEP-1 can address this has been noted briefly in the introduction and “managing expectations and challenges” sections. In summarizing the session, the interventionist offers examples of STEP-1 suggestions, as they might be implemented over the course of a single day with a discussion of potential challenges and strategies to manage them.

#### Relaxation Education (Control Condition) Delivery

The relaxation education is delivered in a single one-on-one synchronous videoconference session. This session lasts 60 minutes and is delivered by a study team member using a slide deck of 23 slides and following a structured outline. A copy of the presentation slides and a curated resource sheet of web-based videos are provided to each control group participant by email. The presentation begins with a brief description of how relaxation may be of benefit for insomnia. Participants are introduced to a variety of common relaxation exercises (eg, progressive muscle relaxation, paced breathing, and guided imagery) and given information about how to access relaxation exercises independently during the study period. Specifically, they are provided access to a free app and given a list of videos available on the web. During the session, participants are given the opportunity to practice accessing relaxation exercises on the app and recommended videos on the web with study staff providing technical support as needed. Participants who do not have a mobile device compatible with the app are loaned a device for the study period. To close the session, the presenter makes suggestions to the participants on how to integrate these exercises into their routines to reduce stress and improve sleep independently. Self-guided online relaxation intervention was selected for the control condition, as 90% of American people use the internet [52], and the internet is very often used by cancer survivors for health information [53]. Delivering this education session in a way that closely follows the structure and methods used in the STEP-1 session allows for control for attention and other nonspecific factors while also addressing ethical and practical problems associated with waitlist or no-treatment controls [54].

#### Follow-Up

After completing their assigned interventions, participants in both conditions are scheduled for their 4-week follow-up call (Figure 1) and are asked about their preference (eg, phone, email, and mail) for reminders.

#### Baseline Session Evaluation

All participants are asked to complete a satisfaction form evaluating their experience with the intervention session upon its conclusion. Participants receive a link to a questionnaire on which they report on ease of use and acceptability, satisfaction, and credibility of the session. Participants are encouraged to complete it while still in the intervention session but have the option to complete it later. Participants receive a US $10 gift card upon completion of this evaluation.

#### Postintervention Follow-Up: Outcome Data Collection

Study staff contact participants by telephone at previously scheduled times, 4 and 8 weeks postbaseline. Participants are asked to enter follow-up data directly into Qualtrics and are provided with technical assistance if needed. If participants cannot complete the full assessment at the scheduled time due to time constraints, they are asked to complete the 7-item ISI (primary outcome measure) verbally, and their responses are collected; they are then asked to complete the full questionnaire at their earliest convenience. Participants receive email reminders in advance of each phone call. At the 4-week call, participants schedule a phone call for the 8-week assessment. Upon completion of the 4- and 8-week assessments, participants receive a US $25 gift card. At the time of their 8-week follow-up phone call, participants are asked if they would be willing to complete an optional additional ISI assessment 2 months later; participants who agree receive an email with a Qualtrics link for this assessment. The link is sent again a week later for nonresponders, but participants receive no additional reminders and do not receive an incentive for this final optional assessment.

#### Coaching Sessions

Coaching sessions have been demonstrated as an effective enhancement to behavioral interventions for cancer survivors and other populations [55-59]. Including them in this trial will allow for collecting data on their potential utility for enhancing the STEP-1 intervention. Participants randomized to the STEP-1 condition who did not have a ≥6-point decrease in their ISI score [60] at 8-week follow-up are offered the option to continue on the study for 4 weeks. During this time, participants will receive 2 individualized coaching sessions to explore the feasibility of delivering individualized coaching sessions. The optimal timing for the first coaching session is within 7 days after the 8-week follow-up, and the second session should be 7 days after the first. However, they may be scheduled any time within 23 days after the 8-week follow-up with at least 5 days between coaching sessions. Paraprofessional coaches trained and supervised by psychologists with CBTI expertise deliver the coaching sessions remotely by following a structured outline adapted from the principal investigator’s (CJR) insomnia study for young adult cancer survivors [34]. During 30-minute coaching sessions, participants review progress and challenges in following their sleep action plan. Coaches offer support and encouragement and may refer participants to the on-hand materials to help answer questions and reinforce program goals and suggestions.

Following principles of symptom self-management [59,61-64], coaching sessions are not prescriptive but promote survivors’ self-management skills (eg, goal-setting and problem-solving) to implement their sleep action plan. At the end of the 4-week period, participants complete a questionnaire assessing acceptability and satisfaction with the coaching sessions and complete the ISI and sleep change measure. Participants will receive a US $25 gift card upon completion of this questionnaire. Administrative data on coaching sessions missed, interrupted, or rescheduled as well as session duration will be collected.

#### Intervention Training and Supervision

##### STEP-1 Intervention

Study interventionists have graduate-level training in social work, psychology, or related disciplines but no prior training in behavioral sleep medicine. Interventionists begin training by familiarizing themselves with the STEP-1 protocol and participant-facing materials and reviewing them with the principal investigator (CJR). Trainees are asked to closely review the STEP-1 presentation slides and the structured outline detailing how each slide should be presented to participants before reviewing audiotaped, videotaped, and transcribed STEP-1 sessions. When possible, and with the consent of a participant, trainees attend at least 1 live intervention session to observe STEP-1 administration. Following the session, the interventionist answers any questions the trainee might have and demonstrates postsession procedures. As a next step, trainees deliver the STEP-1 intervention in mock intervention sessions with confederates playing the role of a study participant; an experienced interventionist acts as the confederate in at least 1 mock session. These mock sessions are recorded and transcribed before being reviewed for fidelity by the principal investigator (CJR). The principal investigator (CJR) approves interventionists to conduct STEP-1 sessions with study participants after they demonstrate fidelity to the structured outline for each slide of the STEP-1 presentation. Additionally, to maintain fidelity over time and reduce deviation between interventionists, interventionists record and transcribe an intervention session (recording only themselves and not the participant) after every 5 to 10 participants; these transcriptions are scored for fidelity and discussed with the principal investigator (CJR).

##### Self-Guided Relaxation Education

Interventionists begin training on the relaxation education intervention by familiarizing themselves with the patient-facing materials. They closely review the presentation slides and structured outline detailing how each slide should be presented. These materials are then discussed with the principal investigator (CJR) and other study team members as a group. As in STEP-1 training, when possible, trainees attend at least 1 live intervention session (with participant consent). The trainee observes session administration, and the interventionist answers any questions the trainee might have and demonstrates postsession procedures. As this condition introduces a self-management resource-based intervention, interventionist training focuses on taking the proper steps to explain the intervention, assisting participants in accessing the online materials, and supporting them in independently selecting exercises they wish to try. Control session interventionists are approved to conduct the relaxation education sessions with study participants after demonstrating reliability following the structured outline for each slide of the presentation in a recorded mock interview reviewed by the principal investigator (CJR).

##### Coaching

Paraprofessionals are trained as STEP-1 coaches using a coaching session outline and semistructured script. Coach trainees listen to a minimum of 3 live coaching sessions conducted by an experienced coach. The trainee and experienced coach discuss the flow of the coaching script and participants’ reports of sleep action plan challenges and successes. Trainees then conduct 2 audiotaped mock coaching sessions with an experienced coach or psychologist with CBTI experience acting as a participant and receive feedback on the content of the sessions. Finally, trainees conduct at least 2 live sessions with study participants accompanied in the session by an experienced coach serving as a session coleader. Upon review of these training procedures, the principal investigator (CJR) approves coaches to conduct coaching sessions with study participants.

### Measures

Outcome measures for this trial are the ISI [65] (primary) measuring the change in insomnia symptoms and the Profile of Mood States Short Form [66] (POMS-SF; secondary) measuring the change in mood symptoms. The 7-item ISI is the most commonly used measure in insomnia research and has been validated in cancer populations [67]. It has demonstrated adequate internal consistency and is sensitive to detect changes in perceived sleep difficulties with treatment. The POMS-SF is a commonly used measure of psychological distress that has been validated in cancer populations [68]. It is a 35-item measure that assesses the mood states of participants and provides multiple scales including an overall Total Mood Disturbance score used here.

Additional information to describe participants’ sleep was collected using the Patient-Reported Outcomes Measurement Information System Sleep Disturbance Short Form [69], the Consensus Sleep Diary-Morning [70], and the Morningness-Eveningness Questionnaire [71]. The 8-item Patient-Reported Outcomes Measurement Information System Sleep Disturbance Short Form evaluates sleep disturbances and their impact on overall sleep quality; items include specific sleep problems (eg, restless sleep) as well as satisfaction with sleep quality and amount of sleep. Nine items from the Consensus Sleep Diary-Morning are used to assess daily sleep quality and sleep latency; items include time of attempts to fall asleep, number and duration of awakenings, time of final awakening, final rise time, and perceived sleep quality. Five items from the Morningness-Eveningness Questionnaire asking about preferred times for sleep, waking, and physical and mental activities are used to evaluate chronotype.

To describe participants’ demographic characteristics, medical history, current health, and program adherence, several additional measures are used. Demographic information (eg, gender and education), medical information (eg, cancer history and chronic conditions), and insomnia history (eg, insomnia duration, burden, and past treatments) are collected by a direct patient report on a baseline survey developed for this study. The survey also includes 1 physical health item from the Short Form Health Survey [72] asking respondents to rate their overall health, as well as a Pain Thermometer asking participants to rate their current pain on a 1-10 scale [73]. The sleep action report gathers information on changes made in sleep behaviors during the study period, including both changes suggested or recommended in the intervention sessions as well as those not included in the study interventions (eg, change in sleep medications and starting a nonstudy behavioral intervention).

Finally, several participant report measures will be used to capture participant experience and satisfaction with the intervention. Ease of use and acceptability of the online videoconference format will be assessed using 14 checklist items adapted from the Telehealth Usability Questionnaire [74], intervention credibility will be assessed using the 6-item credibility or expectancy questionnaire [75], and satisfaction with the interventions (STEP-1 and relaxation education) will be assessed with checklist items adapted from principal investigator’s previous survivorship education trials [30]. At the 8-week postintervention time point, participants will complete a brief questionnaire to provide feedback about the intervention and how it could be improved. Participants who receive the coaching sessions will also complete a brief coaching satisfaction evaluation after the second coaching phone call. Details of all measures and time points are included in Table 2.

**Table 2:** Study measures.

Measure	Baseline	4-Week follow-up	8-Week follow-up	Coaching session #2	16-Week follow-up (optional)
Insomnia Severity Index	✓	✓	✓	✓	✓
Profile of Mood States Short Form	✓	✓	✓		
PROMIS^a^ Sleep Disturbance Short Form	✓		✓		
Consensus Sleep Diary			✓		
Morningness-Eveningness Questionnaire	✓				
Demographic information	✓				
Medical information	✓				
Single item from the Short Form Health Survey	✓	✓	✓		
Pain Thermometer	✓	✓	✓		
Sleep action report (behavioral) or relaxation action report (control)		✓	✓	✓	
Nonstudy sleep treatment change		✓	✓	✓	
Sleep action plan (behavioral)	✓				
Intervention session evaluation	✓				
Telehealth Usability Questionnaire	✓				
Credibility questionnaire	✓				
STEP-1^b^ satisfaction evaluation			✓		
Coaching evaluation				✓	

^a^PROMIS: Patient-Reported Outcomes Measurement Information System.

^b^STEP-1: Sleep Treatment Education Program.

### Statistical Analysis

#### Primary and Secondary Aims

This is a randomized 2-arm trial comparing the STEP-1 intervention (experimental arm) to a self-guided relaxation education intervention (control arm). The primary end point is a change in ISI score from baseline to 8 weeks postintervention. The secondary end point is a change in mood symptoms on the POMS-SF at 8 weeks postintervention. Change scores will be treated as continuous variables. A repeated measures ANOVA [76] will be used to determine if the STEP-1 intervention significantly reduces insomnia symptoms (ISI scores) and improves mood symptoms (POMS-SF scores) compared to a control condition. ISI and POMS-SF scores will be treated as dependent variables using 2-way ANOVA [76] procedures (α=.05) with 1 between-subjects factor (group; STEP-1 vs control), 1 within-subjects factor (time), and an interaction term (group×time). A clinically significant response to the STEP-1 intervention will be defined as a reduction of ISI scores ≥6 points at 8 weeks. Cohen *d* [77] will be used to quantify within-group effect sizes, and Hedges *g* [78] adjustment will estimate between-group effect sizes. ISI and POMS-SF collected at 4 weeks postintervention will be analyzed using similar methods. Secondary aims will be analyzed using descriptive statistics (secondary aims 2, 3, and 4), logistic regression (secondary aim 1), and 2-tailed *t* tests and chi-square analyses (secondary aim 2) [76].

#### Sample Size and Statistical Power for Primary End Point

To meet the primary aims, we require 60 participants with evaluable data but will enroll 70 as a hedge against attrition. As is common for most CBTI interventions [79], STEP-1 has demonstrated large effects of *d*≥1.00 in our pilot-testing [30]. Using this effect size as a reference, the study is conservatively powered to detect an effect size of 0.6 (primary aim). With a sample size of 60 (30 per arm), the study will have 90% power to detect an effect of this size (2-sided test with α=.05). In the event that the sample size is reduced to 50 (25 per arm), the study will retain >80% power to detect an effect of this size (2-sided test with α=.05).

#### Missing Data

The primary analysis will be an intention-to-treat analysis of randomized subjects. We anticipate minimal missing data (<10%) based on experience using similar data collection methods. If needed, we will use multiple imputation to account for missing data. Baseline sleep and mood variables will be included as potential factors in the imputation model. Sensitivity analysis will impute missing change scores with values sampled from an estimated distribution of change scores from the control group participants.

### Ethical Considerations

Prior to study activation and participant enrollment, the study protocol was approved by the Dana-Farber/Harvard Cancer Center Institutional Review Board (protocol 20-516) and registered on ClinicalTrials.gov (NCT05519982). Prior to enrollment in this minimal-risk study, potential participants complete a consent process approved by the Dana-Farber Cancer Institute’s IRB. Before enrolling, potential participants receive the STEP-1 study information sheet, containing all the elements of informed consent. At the beginning of the intervention session, the interventionist reviews the study information sheet and answers any questions the potential participant may have. Additionally, survivors last screened for eligibility more than 2 weeks prior are rescreened for eligibility. Finally, eligible individuals who wish to enroll in the study are asked to provide consent using an online consent process in use at the Dana-Farber Cancer Institute. Participants receive online gift cards to compensate them for the completion of all postsession questionnaires. The value of the gift cards ranges from US $10 to US $25 in accordance with time required to complete the questionnaires. The maximum compensation for any individual participant who completes all postsurvey questionnaires is US $85 in gift cards. All data are securely stored using HIPAA-compliant servers and software approved for the use of protected health information. Additionally, data are deidentified upon collection and stored under a study identification number.

## Results

This trial was funded by the National Cancer Society in July 2022, and the protocol described here was approved in January 2023. Enrollment and data collection began in February 2023; a total of 70 participants were enrolled as of September 2024. Final data collection concluded in October 2024. We anticipate that data analysis will begin in fall 2024, and results are expected in winter 2025.

## Discussion

### Principal Findings

This study is the first randomized controlled trial to test the hypothesis that the STEP-1 intervention significantly reduces insomnia in cancer survivors (aim 1). By comparing change in symptoms in the STEP-1 participants compared to those in the control condition, the trial will help determine if STEP-1 effects go beyond those that could be attributed to placebo and other nonspecific treatment factors. With additional measures and more extended follow-up than in the previous pilot, this trial will also provide more information about how durable STEP-1 effects may be and if improvement is seen in mood symptoms as well as insomnia (aim 2). In addition, exploratory analyses of participant characteristics will focus on identifying subgroups of cancer survivors most likely to benefit from STEP-1 and evaluate the suitability of the relaxation education control condition for the cancer survivor population (secondary aims 1 and 2). Finally, by testing the utility of individualized coaching sessions and eliciting participant suggestions to improve STEP-1, study results will inform future directions for further optimizing the intervention (aims 3 and 4).

### Comparison to Prior Work

Despite numerous clinical trials supporting its effectiveness [23,25-28], CBTI remains woefully underused by cancer survivors due to barriers to access and demands of the treatment itself [19-22,80-82]. To address limited access and adherence to standard CBTI, STEP-1 was designed as a low-intensity behavioral intervention presenting established elements of CBTI in a single online educational format. STEP-1 was adapted from a single-session sleep education intervention previously developed as part of a stepped-care insomnia intervention for survivors [83]. Consistent with the ORBIT model for the development of behavioral interventions [31-33], pretesting with cancer survivors (phase I) guided refinements of STEP-1, and a subsequent single-arm pilot (phase IIa) supported its potential to reduce insomnia in cancer survivors [30]. Though it is informed by CBTI concepts and methods as a single-session educational intervention delivered by nonsleep specialists, STEP-1 represents a significant departure from standard CBTI, which typically requires 5 or more treatment sessions delivered by mental health specialists. Results of this controlled trial are now needed to justify and inform further development and testing of the novel approach taken by STEP-1. Specifically, the results of the current trial will inform the design of these new trials by guiding the selection of participants, control groups, and measures and further optimization of the intervention.

### Strengths and Limitations

Conceptualized within the ORBIT model, this early phase trial (IIb) is not intended to conclusively evaluate the efficacy of STEP-1 but rather to confirm the appropriateness of conducting a future (phase III) effectiveness trial and to inform its design. As such, the current trial design has several notable limitations. Because the primary end point of the study is at 8-week follow-up, it will not shed light on the longer-term effects of the intervention. Similarly, with a modest size sample of English-speaking participants, there will be limited data on how demographic, cultural, and other participant variables moderate the effects of the intervention. Despite these limitations, by including a larger sample, a control condition, and a longer follow-up period, trial data will provide new and clinically relevant information essential to evaluating the efficacy of STEP-1 and the low-intensity self-management approach to insomnia intervention more generally.

### Conclusions

It is well accepted that behavioral treatment significantly reduces insomnia symptoms and should be the first-line treatment for most patients [84,85]. Unfortunately, it is also widely accepted that due to treatment barriers and challenges, most cancer survivors with insomnia never receive this evidence-based care [23,24]. This trial aims to provide cancer survivors access to effective behavioral insomnia treatment by developing and testing a brief, low-intensity intervention that addresses their needs and interests and can be delivered to them wherever they have internet access. If found effective, this approach has the potential to greatly improve access to care for the growing population of survivors whose health and quality of life are negatively impacted by insomnia.

## Appendix

Multimedia Appendix 1 Study information sheet.

## Abbreviations

CBTI: cognitive behavioral therapy for insomnia
HIPAA: Health Insurance Portability and Accountability Act
IRB: institutional review board
ISI: Insomnia Severity Index
ORBIT: Obesity-Related Behavioral Intervention Trials
POMS-SF: Profile of Mood States Short Form
STEP-1: Sleep Treatment Education Program

## Data Availability

The datasets generated and analyzed during this study are available from the corresponding author on reasonable request.

## References

[1] Aziz NM, Rowland JH (2003). Trends and advances in cancer survivorship research: challenge and opportunity. Semin Radiat Oncol.

[2] Baker F, Haffer SC, Denniston M (2003). Health-related quality of life of cancer and noncancer patients in Medicare managed care. Cancer.

[3] Kroenke CH, Rosner B, Chen WY, Kawachi I, Colditz GA, Holmes MD (2004). Functional impact of breast cancer by age at diagnosis. J Clin Oncol.

[4] Colten HR, Altevogt BM, Institute of Medicine Committee on Sleep Medicine and Research (2006). Sleep Disorders and Sleep Deprivation: An Unmet Public Health Problem.

[5] Stein KD, Syrjala KL, Andrykowski MA (2008). Physical and psychological long-term and late effects of cancer. Cancer.

[6] Wickwire EM, Shaya FT, Scharf SM (2016). Health economics of insomnia treatments: the return on investment for a good night's sleep. Sleep Med Rev.

[7] Fullerton DSP (2006). The economic impact of insomnia in managed care: a clearer picture emerges. Am J Manag Care.

[8] Zhou ES, Recklitis CJ (2014). Insomnia in adult survivors of childhood cancer: a report from project REACH. Support Care Cancer.

[9] Savard J, Morin CM (2001). Insomnia in the context of cancer: a review of a neglected problem. J Clin Oncol.

[10] Ohayon MM, Carskadon MA, Guilleminault C, Vitiello MV (2004). Meta-analysis of quantitative sleep parameters from childhood to old age in healthy individuals: developing normative sleep values across the human lifespan. Sleep.

[11] Walsh JK, Üstün TB (1999). Prevalence and health consequences of insomnia. Sleep.

[12] Kaneita Y, Ohida T, Uchiyama M, Takemura S, Kawahara K, Yokoyama E, Miyake T, Harano S, Suzuki K, Fujita T (2006). The relationship between depression and sleep disturbances: a Japanese nationwide general population survey. J Clin Psychiatry.

[13] Thase ME (2005). Correlates and consequences of chronic insomnia. Gen Hosp Psychiatry.

[14] Katz DA, McHorney CA (2002). The relationship between insomnia and health-related quality of life in patients with chronic illness. J Fam Pract.

[15] Walsh JK (2004). Clinical and socioeconomic correlates of insomnia. J Clin Psychiatry.

[16] Riedel BW, Lichstein KL (2000). Insomnia and daytime functioning. Sleep Med Rev.

[17] Buysse DJ, Angst J, Gamma A, Ajdacic V, Eich D, Rössler W (2008). Prevalence, course, and comorbidity of insomnia and depression in young adults. Sleep.

[18] Breslau N, Roth T, Rosenthal L, Andreski P (1996). Sleep disturbance and psychiatric disorders: a longitudinal epidemiological study of young adults. Biol Psychiatry.

[19] Johnson JA, Rash JA, Campbell TS, Savard J, Gehrman PR, Perlis M, Carlson LE, Garland SN (2016). A systematic review and meta-analysis of randomized controlled trials of cognitive behavior therapy for insomnia (CBT-I) in cancer survivors. Sleep Med Rev.

[20] Espie CA, Fleming L, Cassidy J, Samuel L, Taylor LM, White CA, Douglas NJ, Engleman HM, Kelly H, Paul J (2008). Randomized controlled clinical effectiveness trial of cognitive behavior therapy compared with treatment as usual for persistent insomnia in patients with cancer. J Clin Oncol.

[21] Edinger JD, Wohlgemuth WK, Radtke RA, Marsh GR, Quillian RE (2001). Cognitive behavioral therapy for treatment of chronic primary insomnia: a randomized controlled trial. JAMA.

[22] Sivertsen B, Omvik S, Pallesen S, Bjorvatn B, Havik OE, Kvale G, Nielsen GH, Nordhus IH (2006). Cognitive behavioral therapy vs zopiclone for treatment of chronic primary insomnia in older adults: a randomized controlled trial. JAMA.

[23] Savard J, Savard M (2013). Insomnia and cancer: prevalence, nature, and nonpharmacologic treatment. Sleep Med Clin.

[24] Zhou ES, Partridge AH, Syrjala KL, Michaud AL, Recklitis CJ (2017). Evaluation and treatment of insomnia in adult cancer survivorship programs. J Cancer Surviv.

[25] Kraus SS, Rabin LA (2012). Sleep America: managing the crisis of adult chronic insomnia and associated conditions. J Affect Disord.

[26] Thomas A, Grandner M, Nowakowski S, Nesom G, Corbitt C, Perlis ML (2016). Where are the behavioral sleep medicine providers and where are they needed? A geographic assessment. Behav Sleep Med.

[27] Perlis ML, Smith MT, Benson-Jungquist C, Posner DA (2005). Cognitive Behavioral Treatment of Insomnia: A Session-by-Session Guide.

[28] Ong JC, Kuo TF, Manber R (2008). Who is at risk for dropout from group cognitive-behavior therapy for insomnia?. J Psychosom Res.

[29] Bodenheimer T, Lorig K, Holman H, Grumbach K (2002). Patient self-management of chronic disease in primary care. JAMA.

[30] Chevalier LL, Fine E, Sharma A, Zhou ES, Recklitis CJ (2023). Evaluating the Sleep Treatment Education Program (STEP-1): a single-session educational workshop addressing insomnia in cancer survivors. J Psychosoc Oncol.

[31] Czajkowski SM, Powell LH, Adler N, Naar-King S, Reynolds KD, Hunter CM, Laraia B, Olster DH, Perna FM, Peterson JC, Epel E, Boyington JE, Charlson ME (2015). From ideas to efficacy: the ORBIT model for developing behavioral treatments for chronic diseases. Health Psychol.

[32] Walker LO, Czajkowski SM (2019). Designing interventions to improve the health of women using the ORBIT model. J Obstet Gynecol Neonatal Nurs.

[33] Czajkowski S (2021). Translational research. National Cancer Institute.

[34] Michaud AL, Bice B, Miklos E, McCormick K, Medeiros-Nancarrow C, Zhou ES, Recklitis CJ (2023). Sleep Treatment Education Program for Young Adult Cancer Survivors (STEP-YA): protocol for an efficacy trial. JMIR Res Protoc.

[35] Morin CM, Belleville G, Bélanger L, Ivers H (2011). The Insomnia Severity Index: psychometric indicators to detect insomnia cases and evaluate treatment response. Sleep.

[36] Harvey AG (2008). Sleep and circadian rhythms in bipolar disorder: seeking synchrony, harmony, and regulation. Am J Psychiatry.

[37] Fountain NB, Kim JS, Lee SI (1998). Sleep deprivation activates epileptiform discharges independent of the activating effects of sleep. J Clin Neurophysiol.

[38] Smith MT, Perlis ML (2006). Who is a candidate for cognitive-behavioral therapy for insomnia?. Health Psychol.

[39] Morin CM, Espie CA (2003). Insomnia: A Clinical Guide to Assessment and Treatment.

[40] (2011). Your guide to healthy sleep. US Department of Health and Human Services. National Institute of Health.

[41] (2005). Qualtrics homepage. Qualtrics.

[42] (2022). Simple randomization service. Sealed Envelope Ltd.

[43] Zoom for Healthcare. Zoom Video Communications, Inc.

[44] Yenikomshian HA, Lerew TL, Tam M, Mandell SP, Honari SE, Pham TN (2019). Evaluation of burn rounds using telemedicine: perspectives from patients, families, and burn center staff. Telemed J E Health.

[45] Brody JE (2020). The doctor will skype you now: how telemedicine could transform the healthcare sector. The Independent.

[46] Spielman AJ, Caruso LS, Glovinsky PB (1987). A behavioral perspective on insomnia treatment. Psychiatr Clin North Am.

[47] Garland SN, Johnson JA, Savard J, Gehrman P, Perlis M, Carlson L, Campbell T (2014). Sleeping well with cancer: a systematic review of cognitive behavioral therapy for insomnia in cancer patients. Neuropsychiatr Dis Treat.

[48] Pigeon WR (2010). Treatment of adult insomnia with cognitive-behavioral therapy. J Clin Psychol.

[49] Manber R, Friedman L, Siebern AT, Carney C, Edinger J, Epstein D, Haynes P, Pigeon W, Karlin BE (2014). Cognitive behavioral therapy for insomnia in veterans: therapist manual. US Department of Veteran Affairs.

[50] Glasgow RE, Davis CL, Funnell MM, Beck A (2003). Implementing practical interventions to support chronic illness self-management. Jt Comm J Qual Saf.

[51] van Weel-Baumgarten E (2008). Patient-centred information and interventions: tools for lifestyle change? Consequences for medical education. Fam Pract.

[52] Clement J (2021). Share of the offline population of the United States from 2000 to 2021. Statista.

[53] Holmes MM (2019). Why people living with and beyond cancer use the internet. Integr Cancer Ther.

[54] Freedland KE, Mohr DC, Davidson KW, Schwartz JE (2011). Usual and unusual care: existing practice control groups in randomized controlled trials of behavioral interventions. Psychosom Med.

[55] Gell NM, Grover KW, Savard L, Dittus K (2020). Outcomes of a text message, Fitbit, and coaching intervention on physical activity maintenance among cancer survivors: a randomized control pilot trial. J Cancer Surviv.

[56] Altunkurek SZ, Bebis H (2019). The effects of wellness coaching on the wellness and health behaviors of early adolescents. Public Health Nurs.

[57] Berg CJ, Vanderpool RC, Getachew B, Payne JB, Johnson MF, Sandridge Y, Bierhoff J, Le L, Johnson R, Weber A, Patterson A, Dorvil S, Mertens A (2020). A hope-based intervention to address disrupted goal pursuits and quality of life among young adult cancer survivors. J Cancer Educ.

[58] Somayaji D, Blok AC, Hayman LL, Colson Y, Jaklisch M, Cooley ME (2019). Enhancing behavioral change among lung cancer survivors participating in a lifestyle risk reduction intervention: a qualitative study. Support Care Cancer.

[59] Yun YH, Kim YA, Lee MK, Sim JA, Nam BH, Kim S, Lee ES, Noh DY, Lim JY, Kim S, Kim SY, Cho CH, Jung KH, Chun M, Lee SN, Park KH, Park S (2017). A randomized controlled trial of physical activity, dietary habit, and distress management with the Leadership and Coaching for Health (LEACH) program for disease-free cancer survivors. BMC Cancer.

[60] Yang M, Morin CM, Schaefer K, Wallenstein GV (2009). Interpreting score differences in the Insomnia Severity Index: using health-related outcomes to define the minimally important difference. Curr Med Res Opin.

[61] Reb A, Ruel N, Fakih M, Lai L, Salgia R, Ferrell B, Sampath S, Kim JY, Raz DJ, Sun V (2017). Empowering survivors after colorectal and lung cancer treatment: pilot study of a self-management survivorship care planning intervention. Eur J Oncol Nurs.

[62] Foster C, Fenlon D (2011). Recovery and self-management support following primary cancer treatment. Br J Cancer.

[63] Risendal BC, Dwyer A, Seidel RW, Lorig K, Coombs L, Ory MG (2014). Meeting the challenge of cancer survivorship in public health: results from the evaluation of the chronic disease self-management program for cancer survivors. Front Public Health.

[64] McCorkle R, Ercolano E, Lazenby M, Schulman-Green D, Schilling LS, Lorig K, Wagner EH (2011). Self-management: enabling and empowering patients living with cancer as a chronic illness. CA Cancer J Clin.

[65] Bastien CH, Vallières A, Morin CM (2001). Validation of the Insomnia Severity Index as an outcome measure for insomnia research. Sleep Med.

[66] Curran SL, Andrykowski MA, Studts JL (1995). Short Form of the Profile of Mood States (POMS-SF): psychometric information. Psychol Assess.

[67] Savard M, Savard J, Simard S, Ivers H (2005). Empirical validation of the Insomnia Severity Index in cancer patients. Psychooncology.

[68] Baker F, Denniston M, Zabora J, Polland A, Dudley WN (2002). A POMS short form for cancer patients: psychometric and structural evaluation. Psychooncology.

[69] Yu L, Buysse DJ, Germain A, Moul DE, Stover A, Dodds NE, Johnston KL, Pilkonis PA (2011). Development of short forms from the PROMIS™ Sleep Disturbance and Sleep-Related Impairment item banks. Behav Sleep Med.

[70] Carney CE, Buysse DJ, Ancoli-Israel S, Edinger JD, Krystal AD, Lichstein KL, Morin CM (2012). The Consensus Sleep Diary: standardizing prospective sleep self-monitoring. Sleep.

[71] Adan A, Almirall H (1991). Horne & Ostberg Morningness-Eveningness questionnaire: a reduced scale. Person Individ Diff.

[72] Ware JE, Sherbourne CD (1992). The MOS 36-item short-form health survey (SF-36). I. Conceptual framework and item selection. Med Care.

[73] Clinical practice guidelines in oncology: cancer-related fatigue. National Comprehensive Cancer Network.

[74] Parmanto B, Lewis AN, Graham KM, Bertolet MH (2016). Development of the Telehealth Usability Questionnaire (TUQ). Int J Telerehabil.

[75] Devilly GJ, Borkovec TD (2000). Psychometric properties of the credibility/expectancy questionnaire. J Behav Ther Exp Psychiatry.

[76] Tabachnick BG, Fidell LS (1996). Using Multivariate Statistics. 3rd Edition.

[77] Cohen J (1988). Statistical Power Analysis for the Behavioral Sciences. 2nd Edition.

[78] Hedges LV (1981). Distribution theory for Glass's estimator of effect size and related estimators. J Educ Stat.

[79] Edinger JD, Arnedt JT, Bertisch SM, Carney CE, Harrington JJ, Lichstein KL, Sateia MJ, Troxel WM, Zhou ES, Kazmi U, Heald JL, Martin JL (2021). Behavioral and psychological treatments for chronic insomnia disorder in adults: an American Academy of Sleep Medicine systematic review, meta-analysis, and GRADE assessment. J Clin Sleep Med.

[80] van der Zweerde T, Bisdounis L, Kyle SD, Lancee J, van Straten A (2019). Cognitive behavioral therapy for insomnia: a meta-analysis of long-term effects in controlled studies. Sleep Med Rev.

[81] Ma Y, Hall DL, Ngo LH, Liu Q, Bain PA, Yeh GY (2021). Efficacy of cognitive behavioral therapy for insomnia in breast cancer: a meta-analysis. Sleep Med Rev.

[82] Zhou FC, Yang Y, Wang YY, Rao WW, Zhang SF, Zeng LN, Zheng W, Ng CH, Ungvari GS, Zhang L, Xiang YT (2020). Cognitive behavioural therapy for insomnia monotherapy in patients with medical or psychiatric comorbidities: a meta-analysis of randomized controlled trials. Psychiatr Q.

[83] Zhou ES, Michaud AL, Recklitis CJ (2020). Developing efficient and effective behavioral treatment for insomnia in cancer survivors: results of a stepped care trial. Cancer.

[84] Sateia MJ, Buysse DJ, Krystal AD, Neubauer DN, Heald JL (2017). Clinical practice guideline for the pharmacologic treatment of chronic insomnia in adults: an American Academy of Sleep Medicine clinical practice guideline. J Clin Sleep Med.

[85] Qaseem A, Kansagara D, Forciea MA, Cooke M, Denberg TD, Clinical Guidelines Committee of the American College of Physicians (2016). Management of chronic insomnia disorder in adults: a clinical practice guideline from the American College of Physicians. Ann Intern Med.

