# Supplementary material for: Sleep Treatment Education Program for Cancer Survivors: Protocol for an Efficacy Trial
Source: JMIR Res Protoc. 2024 Nov 28;13:e60762. doi: 10.2196/60762 (PMC11638688; doi:10.2196/60762)
Supplement: Multimedia Appendix 1 [file resprot_v13i1e60762_app1.pdf]

# Sleep Treatment Education Program-1 (STEP-1): A Research Study for Cancer Survivors with Insomnia

STEP-1

## What is STEP-1?

STEP-1 is a study for cancer survivors who are experiencing insomnia (difficulty falling or staying asleep). We know that insomnia can be a problem for some cancer survivors. The purpose of this study is to learn if single session online education programs will be useful for improving cancer survivors' sleep.

## What is involved in STEP-1?

Participants in this study will be randomized to either the behavioral education session, which provides information on how to make changes to sleep habits and behaviors, or the relaxation session, which provides an app & resources for relaxation exercises and instructions on how these exercises can help improve sleep. Randomization is like flipping a coin, and you would have an equal chance of being in each session. Study investigators do not know in advance which session you would be in. At the end of the study, you would be offered the chance to participate in the session you did not take part in during the study.

Participation in the study involves:

- Taking part in a one-on-one session using the Zoom videoconference platform, during which you will also complete a brief online questionnaire. This session will take approximately 60-90 minutes. At the end of the session, you will complete a brief questionnaire for which you will receive a $10 gift card.
- Completing brief (about 10 minute) follow-up questionnaires online with a brief phone call 1 and 2 months after the education session. You will receive a $25 gift card upon completing each follow-up questionnaire.
- Some participants may be invited to take part in two individual coaching sessions and complete another questionnaire, and receive another $25 gift card upon completing it.
- Optional: Complete a brief Sleep Checklist 2 months after your last questionnaire.

About 70 people will take part in this study.

No medications are involved in this study.

## You may be eligible if you:

- Are a cancer survivor age 40-89.
- Have had no cancer therapy in the past 4 months or planned in the future.
- Are experiencing sleep problems.

## Are there any risks to being in the study?

It is possible that some people who are randomized to the Behavioral session might experience a brief period of increased fatigue as they make changes to their sleep, but this typically resolves after a short time. Confidentiality can be a risk with any research study, but we take precautions to protect your privacy, as described below.

## Are there any benefits to being in the study?

Some people may experience improved sleep after participating in the study, but others may not. We hope the information learned from this study will lead to effective insomnia treatment to help cancer survivors in the future.

## What about confidentiality?

Your responses to the questionnaires will not be shared with anyone, and will be stored only by a study ID number. In addition, the Department of Health and Human Services has issued a Certificate of Confidentiality insuring the study team cannot be forced to disclose information that could identify participants in civil, criminal, administrative, legislative or other proceedings. The results of this research study may be published and you will not be identified in publications. A description of this clinical trial will be available on http://www.ClinicalTrials.gov as required by U.S. law. This website will not include information that can identify you. At most, the website will include a summary of the results. You can search this website at any time.

## How do I join or what if I have questions?

You can email or call the contacts listed below and a study coordinator will get in touch with you. You will be asked a few questions to determine whether you are eligible for this study. You can contact the Principal Investigator, Christopher Recklitis, PhD at (617) 582-8260. Participation is completely voluntary, and you can choose to stop being in the study or not answer specific questions at any time.

STEP@dfci.harvard.edu (617) 582-8260
STEPforsleep.com
